# Hazelnut oral immunotherapy in children: An Italian single‐center retrospective cohort study

**DOI:** 10.1111/pai.70287

**Published:** 2026-02-23

**Authors:** Simona Barni, Benedetta Pessina, Serena Della Femina, Francesco Catamerò, Claudia Valleriani, Mattia Giovannini, Giulia Liccioli, Lucrezia Sarti, Leonardo Tomei, Francesca Mori

**Affiliations:** ^1^ Allergy Unit Meyer Children's Hospital IRCCS Florence Italy; ^2^ Department of Health Sciences University of Florence Florence Italy; ^3^ Immunology Laboratory Meyer Children's Hospital IRCCS Florence Italy

**Keywords:** anaphylaxis, children, food allergy, hazelnut, IgE, oral immunotherapy

## Abstract

**Introduction:**

Hazelnut oral immunotherapy (H‐OIT) is a promising treatment for hazelnut allergy (HA). The aim of this study was to assess the safety and efficacy of H‐OIT in a pediatric population, while describing its clinical and allergy characteristics.

**Methods:**

Children undergoing H‐OIT at our tertiary pediatric hospital Allergy Unit between April 2015 and December 2024 were enrolled. Demographic and clinical features, allergy test results and information on H‐OIT were recorded.

**Results:**

124 patients (58.1% male; median age 8.5 years) were enrolled, 73 (58.9%) aged <4 years. The most common presenting symptom was urticaria/angioedema (75, 60.5%), followed by anaphylaxis (54, 43.5%). 54/124 (43.5%) reached the protocol's target maintenance dose within a median time of 24 months, 31/124 (25.0%) remained on a lower, partial maintenance dose at a median tolerated dose of 1000 mg of ground hazelnut. The treatment discontinuation rate was 31.5%, with over half (53.9%) due to loss to follow‐up. 102/124 (82.3%) developed a reaction during H‐OIT (72.5% resolved spontaneously, 14.7% occurred at home), of which 9 anaphylaxis (8.8%); only one required adrenaline, and two occurred at home because of a cofactor. Statistically significant reductions in the prick‐by‐prick (PbP) test diameter (*p* < 0.001) and Cor a 14 IgE levels (*p* = 0.004) were observed. A positive family history of atopy was associated with lower odds of completing H‐OIT (odds ratio 0.221, 95% confidence interval 0.055–0.895, *p* = 0.034).

**Conclusion:**

H‐OIT may be an effective treatment option for children with HA despite the high discontinuation rate. One quarter of patients remained on a lower, partial maintenance dose. Although the overall safety profile of H‐OIT seems acceptable, anaphylactic events represent a significant burden for families and must be carefully considered when weighing the risks and benefits of OIT.

**Key message**

Hazelnut avoidance is the gold standard treatment for hazelnut allergy. However, new approaches, including oral immunotherapy, are promising. This retrospective study analyzed a cohort of Italian children allergic to hazelnut who underwent hazelnut oral immunotherapy and showed that it is a procedure with appreciable efficacy. Although the overall safety profile of H‐OIT seems acceptable, anaphylactic events represent a significant burden for families and must be carefully considered when weighing the risks and the benefits of OIT. Moreover, a reduction in the prick‐by‐prick test diameter and Cor a 14 levels after treatment was noted, and a positive family history of atopy was identified as a possible influencer on the success of H‐OIT.

## 1 INTRODUCTION

Hazelnut allergy (HA) is the most prevalent tree nut allergy in Europe, with a reported prevalence of 0.04% in the general population based on the oral food challenge (OFC).^1^ Specifically, in Italy, tree nuts rank as the second leading cause of food‐induced anaphylaxis following cow's milk,^2^ with hazelnuts being the most frequent trigger among all tree nuts.^3^

Currently, the treatment of tree nut allergies, including HA, consists of a strict elimination diet and the use of emergency medications in case of allergic reaction.^4^, ^5^ Although current international guidelines^6^ recommend oral immunotherapy (OIT) only for children over 4 years of age with persistent cow's milk, hen's egg or peanut allergy, some studies suggest that hazelnut oral immunotherapy (H‐OIT) is a promising, safe and effective approach in children.^7^, ^8^, ^9^, ^10^

Clinically, HA can manifest as either a primary nut allergy or pollen food syndrome (PFS). Primary HA is typically associated with systemic and severe reactions driven by serum‐specific IgE (s‐IgE) against major hazelnut storage proteins (e.g., Cor a 9, Cor a 14). In contrast, PFS is usually characterized by mild, localized oropharyngeal signs and symptoms (e.g., oral allergy syndrome, OAS) and occurs in individuals with seasonal allergic rhinitis.^11^ This reaction is caused by s‐IgE targeting heat‐labile proteins (e.g., PR‐10) that are homologous to pollen allergens.^12^, ^13^ A primary nut allergy is more likely in younger children; however, with increasing age, the probability of developing PFS increases.^14^, ^15^ For the same reason, it is more common to be allergic to a single tree nut below the age of two, whereas the likelihood of developing co‐sensitization to multiple tree nuts increases with age.^16^, ^17^, ^18^, ^19^, ^20^, ^21^ We offer H‐OIT to patients with allergy to the major hazelnut storage proteins, namely those with primary hazelnut allergy. Our hypothesis is that these patients tend to be younger, require a long period to complete desensitization, and are more likely to experience severe allergic reactions.

The primary objective of this study was to assess the feasibility, safety, and efficacy of H‐OIT by using hazelnut in an Italian pediatric population with HA, while describing its clinical and allergy characteristics.

## 2 METHODS

### 2.1 Study design

In this single‐center retrospective cohort study, children undergoing H‐OIT at the Allergy Unit of Meyer Children's Hospital IRCCS, Florence, Italy from April 2015 to December 2024, were enrolled. Inclusion criteria were (1) a confirmed diagnosis of IgE‐mediated HA and (2) consent to undergo H‐OIT. The diagnosis of HA was confirmed using the following criteria: (1) history of anaphylaxis to hazelnut and positive PbP and/or s‐IgE to hazelnut or, in the absence of history of anaphylaxis, (2) positive hazelnut open, unblinded OFC following the protocol described by Moraly et al.^7^ Exclusion criteria were any contraindications to undergo OIT according to the current guidelines.^22^

Demographic and clinical features, allergy tests results, co‐allergies, comorbidities and information about H‐OIT were recorded.

Electronic medical charts were reviewed and data such as sex (female/male), age (in months), family history of atopy (present/absent), presence/absence of atopic comorbidities (asthma, allergic oculorhinitis, atopic dermatitis, and other food allergies), and history of reactions to hazelnut (clinical signs and symptoms, treatment) were collected.

Skin prick tests (SPTs) for the main pollen allergens (grass, pellitory, mugwort, cypress, olive tree, hazel tree, birch pollen, and poplar tree) and prick‐by‐prick (PbP) tests for hazelnuts were performed and considered positive if the wheal diameter was ≥ 3 mm at the 15 min reading. Histamine (10 mg/mL; Lofarma, Milan, Italy) and normal saline were used as the positive and negative controls, respectively. PbP tests are performed by piercing the fresh hazelnut with a sterile lancet and then immediately applying the lancet to the patient's skin, following standard allergy testing procedure as explained in Tagliati et al.^3^ Hazelnut s‐IgE and Cor a 1, Cor a 8, Cor a 9, and Cor a 14 s‐IgE were determined using a commercial assay (ImmunoCAP system, Thermo Fisher Scientific, Uppsala, Sweden) with a positive cut‐off point set at 0.1 kUA/L.

### 2.2 H‐OIT protocol

According to standard practice in our Allergy Unit, H‐OIT was proposed to all patients with the abovementioned criteria and was initiated in Day Hospital regimen in the Allergy Unit of our Hospital.

In summary, an initial dose of 5 mg of hazelnut (corresponding to 0.75 mg of hazelnut proteins) was administered and then increased every 30 min according to the following scheme: 5 + 10 + 50 + 100 + 200 + 400 + 600 + 900 + 1500 + 3000 + 5000 mg of hazelnut if the patient shows no reactions. All doses administered and referred to throughout this manuscript are presented as the weight of fresh ground hazelnut, unless otherwise specified. If any objective skin, respiratory, and/or cardiovascular, neurologic or gastrointestinal clinical manifestations were observed, patients were promptly treated and advised to continue treatment at home with the last tolerated dose at the OFC. The H‐OIT protocol consists of subsequent Day Hospital visits every about 3–6 months to increase the dose. Between visits, patients were instructed to take the dose at least 3 times/week after a meal at home, to avoid physical activity in the hour before and in the 3 hours after hazelnut ingestion, and to halve the dose in case of acute intercurrent diseases. Patients able to tolerate the final OFC step of 5000 mg of hazelnut, with a cumulative challenge dose of 11,765 mg of hazelnut (roughly 8 hazelnuts, corresponding to 1764.75 mg of hazelnut proteins), were considered desensitized. Patients who successfully reach the final single OIT dose of 5000 mg of hazelnut are not allowed to consume hazelnuts ad libitum. Instead, they are instructed to continue ingesting this specific dose at least three times per week, under controlled conditions (e.g., after meals, avoiding exercise before and after ingestion…). In patients who discontinued OIT, we recommended consumption of food products containing hazelnut traces (as indicated by precautionary allergen labelling) unless the patient showed reactions to such products.

Informed consent/assent was obtained from parents/legal guardians and children when appropriate for all procedures. This study was approved by the Ethic Committee Tuscany Region – Pediatric (Reference #195/2025).

### 2.3 Statistical analysis

Statistical analyses were performed using the IBM Statistical Package for Social Science software (SPSS, Version 28.0, Chicago, IL, USA). No formal power analysis was performed because the study included all eligible patients within the predefined timeframe, making the sample size determined by data availability rather than prospective calculation. Non‐normally distributed variables were presented as medians, while categorical variables were presented as percentages. Continuous variables were compared using the nonparametric Wilcoxon rank‐sum test, whereas categorical variables were compared using the chi‐square test. To identify the factors associated with OIT and OIT success, we performed logistic regression analysis using the occurrence of reactions during OIT and the finalization of OIT as outcome (dependent) variables. Univariate analyses identified associated independent variables for the multivariate model fitted using a backward elimination procedure (likelihood ratio was used as a criterion and *p* > 0.2 for removal). The Hosmer–Lemeshow test was used to evaluate the goodness of fit of the multivariable model. The results are expressed as odds ratios (ORs) and 95% confidence intervals (CI). All statistical tests were two‐sided, and statistical significance was set at *p* < 0.05.

## 3 RESULTS

### 3.1 Baseline characteristics

**FIGURE 1:**
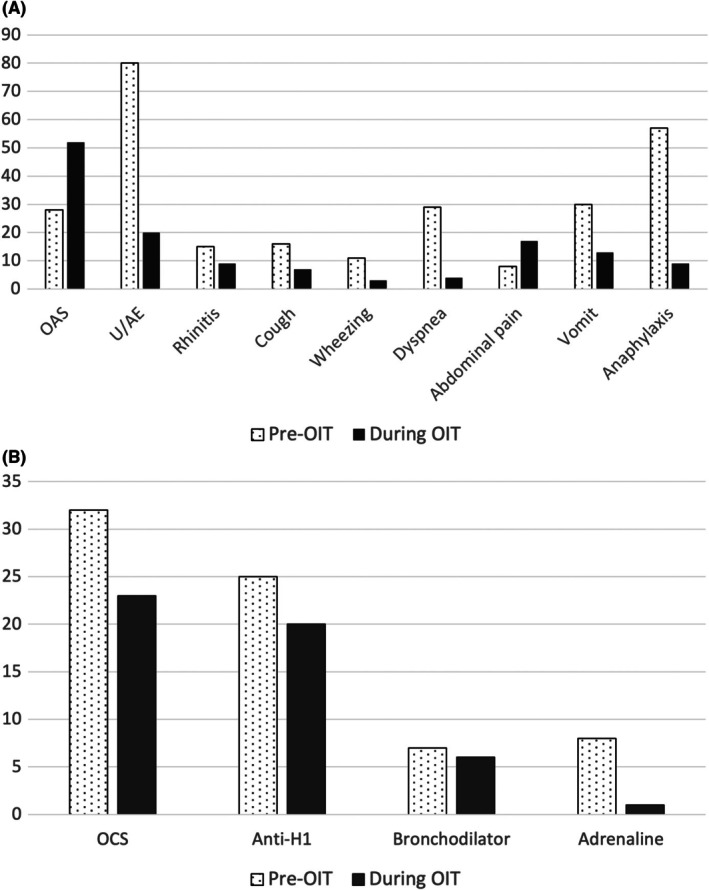
(A) Clinical signs and symptoms frequency upon first allergic reaction to hazelnut and during OIT (both during hospital up‐dosing and at home maintaining phase). (B) Adopted treatment for first allergic reaction to hazelnut and for reactions during OIT. Anti‐H1, antihistamine; OAS, oral allergy syndrome; OCS, oral corticosteroid; OIT, oral immunotherapy; U/AE, urticaria/angioedema.

A total of 124 patients who underwent H‐OIT were enrolled in this study. Of these, 72/124 (58.1%) were male, with a median age at first reaction of 36 months (IQR 24–71) and a median age at the beginning of H‐OIT of 105 months (IQR 70.5–133.5). 73/124 (58.9%) patients were <4 years at the time of reaction, while only 19/124 (15.3%) at the start of H‐OIT. A family history of atopy was present in 101/124 (81.5%) patients; atopic comorbidities were common: atopic dermatitis in 48/124 (38.7%), allergic rhinitis in 52/124 (41.9%) and asthma in 49/124 (39.5%) patients. The demographic characteristics are shown in Table 1. The rate of co‐sensitization to other tree nuts and peanut was high, with 60/124 (48.4%) showing co‐sensitization or co‐allergy to at least one other nut. Nine out of 124 patients (7.3%) were merely co‐sensitized, whereas 51/124 (41.1%) had a history of allergic reactions to other tree nuts. Among the co‐allergic patients, 9/51 were allergic to two other tree nuts and 4/51 were allergic to at least three other tree nuts; the most common co‐allergy was walnuts (37/51, 72.5%) (Table 2). A clinical history of anaphylaxis to hazelnuts was reported in 54/124 children (43.5%), while the most common reaction at onset was urticaria/angioedema (75/124, 60.5%) (Figure 1).

**TABLE 1:** Baseline demographic characteristics and pollen co‐sensitization frequency.

Characteristic	Total *N*	*N* (%)	Median	IQR
Age at reaction (months)	124		36	24–71
Sex, female	124	52 (41.9%)		
Family history of atopy	124	101 (81.5%)		
Atopic dermatitis	124	48 (38.7%)		
Allergic rhinitis	124	52 (41.9%)		
Asthma	124	49 (39.5%)		
Age at H‐OIT (months)	124		105	70.5–133.5
Pollen co‐sensitization	124	102		
Grass pollen	102	70 (68.6%)		
Pellitory	102	23 (22.5%)		
Mugwort	102	23 (22.5%)		
Cypress	102	34 (33.3%)		
Olive tree	102	54 (53.9%)		
Hazel tree	102	38 (37.3%)		
Birch	102	50 (49.0%)		
Poplar tree	102	18 (17.6%)		

Abbreviations: IQR, interquartile range; *N*, number; H‐OIT, hazelnut oral immunotherapy.

**TABLE 2:** Co‐allergy and co‐sensitization to other tree nuts and peanut in our cohort. The percentages are calculated on the number of co‐allergic or co‐sensitized patients (60/124).

Tree nuts and peanut	Co‐allergy (*N* = 51/60)	Rate of co‐allergy in the population	Co‐sensitization (*N* = 9/60)	Rate of co‐sensitization in the population
Walnut	37 (72.5%)	37/124 (29.8%)	8 (88.9%)	8/124 (6.5%)
Pistachio	9 (17.6%)	9/124 (7.3%)	2 (22.2%)	2/124 (1.6%)
Cashew	7 (13.7%)	7/124 (5.6%)	2 (22.2%)	2/124 (1.6%)
Peanut	6 (11.8%)	6/124 (4.8%)	3 (33.3%)	3/124 (2.4%)
Almond	4 (7.8%)	4/124 (3.2%)	2 (22.2%)	2/124 (1.6%)
Pine nut	3 (5.9%)	3/124 (2.4%)	2 (22.2%)	2/124 (1.6%)

Abbreviation: *N*, number.

When divided according to age group, children aged <4 years more often presented with vomiting at onset (*p* = 0.029), whereas those aged ≥4 years showed oral allergy syndrome (OAS) (*p* < 0.001) or dyspnea (*p* = 0.010) (Table 3). No significant differences were observed in Cor a 1 (hazelnut PR‐10), Cor a 8, Cor a 9 and Cor a 14 IgE levels in patients with or without OAS/anaphylaxis or in young versus older patients. Treatment for the first reaction was mainly with oral corticosteroids (32/90, 35.6%), oral antihistamines (25/90, 27.8%), or inhaled bronchodilators (7/90, 7.8%), whereas only 8/54 (14.8%) patients with anaphylaxis were treated with intramuscular adrenaline (Figure 1). For 34/124 patients, information about treatment of the first reaction was not present in the medical chart. Patients without a history of anaphylaxis experienced a reaction at the OFC with a median reaction dose of 150 mg of hazelnut (IQR 50–600). Baseline allergy test results are presented in Table 4.

**TABLE 3:** Clinical history of reaction to hazelnut. For 14 patients, information about the initial clinical symptoms was not detailed in the electronic medical chart.

Clinical symptom	Total *N*	Age group class		*p*‐value
		<4 years (*N* = 73)	≥4 years (*N* = 37)	
		*N* (%)	*N* (%)	
OAS	110	9 (12.3)	16 (43.2)	<0.001
U/A	110	51 (69.9)	24 (64.9)	0.595
Rhinitis	110	10 (13.7)	5 (23.5)	0.979
Dyspnea	110	13 (17.8)	15 (40.5)	0.010
Wheezing	110	5 (6.8)	6 (16.2)	0.122
Cough	110	11 (15.1)	5 (13.5)	0.827
Abdominal pain	110	3 (4.1)	4 (10.8)	0.174
Vomiting	110	24 (32.9)	5 (13.5)	0.029
Diarrhea	110	1 (1.4)	0 (0)	0.474
Anaphylaxis	110	36 (45.6)	18 (47.4)	0.855

Abbreviations: *N*, number; OAS, oral allergy syndrome; U/A, urticaria/angioedema.

**TABLE 4:** Baseline allergy tests compared to post H‐OIT allergy tests.

	*N* of patients with positive value pre‐OIT (%)	Pre‐OIT	Post‐OIT	*p*‐value
PbP at start (mm), median (IQR)	124 (100)	6 (5–9)	3.5 (2–5)	<0.001
s‐IgE hazelnut (kU/L), median (IQR)	116 (93.5)	7.06 (2.71–25.8)	4.56 (2.3–11.5)	0.113
Cor a 1 (kU/L), median (IQR)	36 (29.0)	0.04 (0–2.11)	0.04 (0.02–7.98)	0.099
Cor a 8 (kU/L), median (IQR)	24 (19.4)	0.04 (0–0.34)	0.14 (0.01–0.41)	0.872
Cor a 9 (kU/L), median (IQR)	77 (62.1)	1.90 (0.32–9.31)	0.56 (0.07–1.43)	0.058
Cor a 14 (kU/L), median (IQR)	84 (67.7)	3.37 (0.8–11.5)	0.38 (0.09–2)	0.006

Abbreviations: IQR, interquartile range; mm, millimeters; *N*, number; OIT, oral immunotherapy; PbP, prick‐by‐prick test; s‐IgE, serum‐specific IgE.

### 3.2 H‐OIT results

H‐OIT was initiated with a median latency of 50 months (IQR 20–93) since the first reported reaction. Of the 124 patients enrolled for H‐OIT, 54/124 (43.5%) reached the protocol's target maintenance dose with a median duration of 24 months (IQR 11–41), 31/124 (25.0%) remained on a lower, partial maintenance dose with a median tolerated dose of 1000 mg of hazelnut (corresponding to 150 mg of hazelnut proteins). The median starting dose was 10 mg of hazelnut (corresponding to 1.5 mg of hazelnut protein). Thirty‐nine out of 124 patients (31.5%) discontinued H‐OIT. The primary reasons were loss to follow‐up (21/39, 53.9%), adverse reactions (8/39, 20.5%), taste aversion (5/39, 12.8%), satisfaction with trace consumption (5/39, 12.8%) and family reasons (2/39, 5.1%). In patients who achieved desensitization, allergy tests were repeated at the end of the treatment and a significant reduction was observed in PbP median diameter (3.5 mm, IQR 2–5, *p* < 0.001) and Cor a 14 (0.38 kU/L, IQR 0.09–2, *p* = 0.006) (Table 4). No significant changes were observed in s‐IgE, Cor a 1, Cor a 9 or Cor a 8 levels pre‐ and post‐H‐OIT. H‐OIT duration did not correlate with any of these allergy tests, but Spearman correlation analysis showed that a weak negative correlation existed between H‐OIT duration and younger age (−0.272, *p* = 0.024).

### 3.3 Adverse reactions

Among the enrolled patients, 102/124 (82.3%) showed at least one adverse reaction, of which only 15/102 (14.7%) were at home. Reactions were mainly localized and self‐limiting, with 52/102 (50.9%) being oral symptoms. Only 28/102 (27.5%) required medical treatment, mainly oral corticosteroids and antihistamine therapy (Figure 1). However, 9/102 patients experienced anaphylaxis (8.8%), two of which were at home; all other anaphylactic episodes occurred at dose increases at the hospital under medical supervision, and only one of them was treated with intramuscular adrenaline. Both anaphylactic episodes occurring outside the hospital were associated with the presence of a cofactor (physical exercise in both cases) and were managed by the parents with oral corticosteroids, antihistamines, and inhaled bronchodilator therapy. One of the two patients who showed anaphylaxis at home finally interrupted OIT. Other common adverse reactions were urticaria (20/102, 19.6%), abdominal pain (17/102, 16.7%), and vomiting (13/102, 12.7%), whereas the less common symptoms were rhinitis (9/102, 8.8%), cough (7/102, 6.8%), dyspnea (4/102, 3.9%), and wheezing (3/102, 2.9%).

### 3.4 Baseline characteristics associated with desensitization/reactions

In the univariate analysis, successful desensitization was associated with female sex, negative family history of atopy, lower baseline PbP, and lower baseline hazelnut s‐IgE levels. In the multivariate analysis, only a family history of atopy remained associated with the outcome of desensitization; in particular, patients with a positive family history had a 20% probability of completing H‐OIT compared with patients without a family history (OR 0.221, CI 0.055–0.895, *p* = 0.034) (Table 5). When assessing the risk of reactions during H‐OIT, no variables, including age, sex, atopic comorbidities, and baseline allergy tests, were associated with the occurrence of reactions.

**TABLE 5:** Baseline variables associated with successful desensitization in univariate and multivariate analysis.

Variable	Odds ratio (95% CI)	
	Univariate analysis	Multivariate analysis
Age at beginning	0.997 (0.989–1007)	
Female sex	2.000 (0.852–4.694) *p*‐value 0.111	2.309 (0.914–5.833) *p*‐value 0.077
Family history of atopy	0.238 (0.063–0.896) *p*‐value 0.034	0.221 (0.055–0.895) *p*‐value 0.034
Atopic dermatitis	0.941 (0.403–2.200)	
Asthma	0.835 (0.395–2.120)	
Allergic rhinitis	0.960 (0.418–2.205)	
Co‐allergy with other tree nuts and/or peanut	1.316 (0.576–3.006)	
Baseline PbP wheal diameter	0.879 (0.755–1.023) *p*‐value 0.097	1.154 (0.974–1.367) *p*‐value 0.098
Hazelnut s‐IgE (*n* = 114)	0.988 (0.972–1.005) *p*‐value 0.163	1.007 (0.989–1.024) *p*‐value 0.450
History of anaphylaxis	1.618 (0.705–3.709)	
Latency from reaction to OIT (time in months)	0.994 (0.985–1.004)	

Abbreviations: CI, confidence intervals; OIT, oral immunotherapy; PbP, prick‐by‐prick test; s‐IgE, serum‐specific IgE.

## 4 DISCUSSION

To the best of our knowledge, this is the first Italian retrospective study aimed to assess the safety and efficacy of H‐OIT and the largest one in literature,^7^, ^8^, ^9^ which comprises retrospective studies on pediatric French populations and only one Israeli prospective study.^10^

Comparisons of our efficacy and safety outcomes with those reported in the other published studies are challenging, owing to fundamental differences in study design, population characteristics, and, importantly, the definition of desensitization. Table S1 provides an overview of the key characteristics of the studies published in the literature so far.

In our study, H‐OIT proved efficacious in achieving desensitization. 43.5% of the patients were fully desensitized, which is slightly higher than what was reported by Moraly et al. (34%)^7^ but lower than that of Sabouraud (51.4%),^8^ Casanovas (52.2% in intention‐to‐treat analysis and 76.7% in per protocol analysis),^9^ and Elizur (96.7%),^10^ due to several factors. First, the high protein maintenance dose: recent studies have demonstrated that low‐dose OIT is still efficacious for different allergens and shows an improved safety profile.^23^, ^24^, ^25^, ^26^, ^27^ Second, the high dropout rate in our real‐world study, primarily due to loss to follow‐up. This is likely influenced by the long study period (9 years), which also included the COVID‐19 pandemic period, and could also be due to long up‐dosing intervals. Our results are consistent with those of Casanovas et al.^9^ where 37.5% of the patients discontinued desensitization, and 19.3% were lost to follow‐up. In contrast, the dropout rate was lower in the study by Sabouraud et al. (21.4%)^8^ and the main reason for discontinuation was hazelnut aversion (5/15, 33.3%). We also identified hazelnut aversion as a cause of dropping out in a lower percentage of patients (5/39, 12.8%). Roughly a quarter of our cohort remained on a lower, partial maintenance dose tolerating a median dose of 150 mg of hazelnut protein, corresponding to 100‐fold the starting dose. Although protection from accidental exposure cannot be guaranteed at this stage, previous studies have shown that even low doses—such as 68 mg or 300 mg of hazelnut protein—may confer partial protection and desensitization in most patients.^8^, ^28^ These individuals will require continued follow‐up and dose escalation to achieve more robust and sustained clinical tolerance.

The efficacy of H‐OIT can also be observed from the lower incidence of anaphylaxis during H‐OIT (8.8%) compared to the rate observed during the first hazelnut clinical reaction (43.5%), paralleled by a statistically significant reduction in PbP median diameter and Cor a 14 levels before and after H‐OIT, replicating results by Elizur et al.^10^ At present, these biomarkers may be useful for tracking immunological trends, but further research is needed to identify clinically actionable thresholds to guide desensitization monitoring.

Our desensitization rate may also be influenced by the age at which hazelnut desensitization was started. The average age at the start of H‐OIT was approximately 9 years, as reported in other studies,^7^, ^8^, ^9^ although the average age of the first reaction to hazelnut was 3 years. This prolonged latency is partly due to the hospital's waiting lists and delayed referral by primary care pediatricians, who tend to postpone referring patients due to current international guidelines not recommending OIT for tree nuts.^6^ This trend is likely to change thanks to recent evidence showing that in children with IgE‐mediated peanut allergy, the earlier the desensitization starts, the higher the chance of sustained unresponsiveness.^29^

The average H‐OIT duration was 2 years in our cohort, and we demonstrated a weak inverse association between age and H‐OIT duration; thus, younger children terminated OIT in a longer timeframe. Younger children may be more likely to have a primary tree nut allergy, leading to a slower dose increase, or may be more prone to infections, leading to temporary dose reductions. The long duration may again be dependent on waiting list problems, allowing to provide up‐dosing visits every 3–6 months only.

Regarding safety, most patients showed some reaction during OIT, but most of these were mild: 82.3% of patients experienced reactions, compared to 30%–57.1% of patients described in other studies.^7^, ^8^, ^9^ Elizur et al. showed a 70% reaction rate during up‐dosing and 63.3% at home.^10^ However, most reactions in our study were local (e.g., OAS), and only 30% required treatment. However, nine patients (8.8%) experienced anaphylaxis during OIT, 22.2% (2/9) at home, always in association with cofactors, which are known to reduce tolerance thresholds.^30^, ^31^ Considering the anaphylaxes occurred without cofactors, 6.9% of our patients (7/102) experienced anaphylaxis. Moraly et al.^7^ report a lower rate of anaphylaxis probably due to the fact that 20% of the patients were only sensitized to hazelnuts (based on allergy tests performed for atopic dermatitis), whereas none of our patients was merely sensitized, 43% showing anaphylaxis to hazelnuts as the first reaction. Moreover, the maintenance dose of hazelnut protein we used (1764 mg) was higher than that of Moraly et al. (416 mg).^7^ The percentage of patients who experienced anaphylaxis during H‐OIT in our cohort was also higher than that reported by Sabouraud et al.,^8^ who showed that only 2.9% experienced severe systemic allergic reactions, with one patient requiring an adrenaline autoinjector at home and whose maintenance dose was individualized. It can be inferred that the median cumulative ingested dose at the initial consultation, at 6 months, and at the 1‐year follow‐up was 21.4 mg, 68.3 mg, and 220.9 mg of hazelnut protein, respectively, so that the higher frequency of anaphylaxis reported in our study may again be explained by the higher maintenance dose. In contrast, Casanovas et al. used a cumulative dose of 1490 mg of hazelnut protein at the second OFC to consider a patient desensitized,^9^ similar to our maintenance dose, which may explain why our results are more consistent, with 7.4% of patients experiencing anaphylaxis and 2.6% requiring adrenaline at home. A similar rate of anaphylactic reactions is reported also by Elizur et al. 13.3% and 16.7% treated with adrenaline during up‐dosing and at home, despite the lower maintenance dose of 1200 mg of hazelnut protein.^10^

In our study, among the seven patients who experienced anaphylaxis in the hospital setting, only one was treated with adrenaline, while none of the reactions occurring at home were managed with adrenaline. This highlights a well‐recognized issue: although international guidelines recommend adrenaline as a first‐line treatment for anaphylaxis,^32^, ^33^ it is still underutilized by both medical and non‐medical personnel.^22^, ^34^, ^35^, ^36^, ^37^, ^38^, ^39^, ^40^ Several factors contribute to the failure to administer adrenaline, including misdiagnosis of the reaction and concerns about potential adverse effects. Educational and training programmes for both medical and non‐medical personnel are currently being explored to increase the appropriate use of adrenaline.^41^

In our study, multivariate analysis showed that a family history of atopic disease was inversely proportional to the likelihood of completing desensitization. Only 20% of patients with a family history of atopy completed the treatment. This may be due to the caregivers' awareness or fear of possible allergic reactions upon ingestion of the trigger food.^42^

All these data underline the need for careful patient selection before starting OIT, discussing the risk–benefit ratio with the patient and the family, and the importance of compliance. However, these findings are currently not sufficient to guide risk‐stratified patient selection or management decisions. Future studies are needed to develop and validate predictive models that could support personalized OIT strategies.

This is the first Italian study to investigate the efficacy and safety of H‐OIT in a pediatric population. Second, this is the largest study to date that has analyzed 124 hazelnut‐allergic patients enrolled in H‐OIT. Third, the long enrollment period (9 years). Finally, this study reflected the real‐world experience of how children with HA are treated at a tertiary care hospital in Italy.

However, this study also shows several limitations. First, this was a single‐center study; therefore, the findings may not apply to other populations. Second, it had a retrospective design with a risk of recall bias, missing data and changes in clinical practice in the long follow‐up period. The third limitation is the absence of a control group: without a randomized controlled trial, it is difficult to accurately compare the effectiveness of H‐OIT with other management strategies. However, this is a limitation common to all published studies. Finally, another limitation is that our study did not use a double‐blind, placebo‐controlled OFC to confirm HA, although we performed an open OFC in patients who had not experienced anaphylaxis to hazelnut in their clinical history. Another notable limitation of our study is the relatively high discontinuation rate (31.5%), largely due to loss to follow‐up rather than treatment‐related adverse events. This highlights the challenges of long‐term adherence in real‐world OIT programs and suggests the need for improved strategies to maintain patient engagement throughout the treatment course (e.g. psychological support, reduced waiting lists, telemedicine follow‐up tools…). Moreover, we did not assess sustained unresponsiveness at the end of desensitization, and appropriately designed studies are required for this purpose.

In conclusion, H‐OIT may be an effective treatment option for children with HA despite the high discontinuation rate. One quarter of patients continue with a lower, partial maintenance dose. Although the overall safety profile of H‐OIT seems acceptable, anaphylactic events represent a significant burden for families and must be carefully considered when weighing the risks and the benefits of OIT. H‐OIT may be offered to selected children with hazelnut allergy when the risks and the benefits are carefully considered.

## AUTHOR CONTRIBUTIONS

**Simona Barni:** Conceptualization; project administration; writing – original draft; writing – review and editing; investigation. **Benedetta Pessina:** Writing – original draft; writing – review and editing; methodology; formal analysis; investigation; conceptualization. **Serena Della Femina:** Data curation; writing – review and editing; writing – original draft. **Francesco Catamerò:** Writing – review and editing; investigation; data curation. **Claudia Valleriani:** Methodology; investigation; writing – review and editing. **Mattia Giovannini:** Visualization; validation; investigation; writing – review and editing. **Giulia Liccioli:** Visualization; validation; investigation; writing – review and editing. **Lucrezia Sarti:** Visualization; validation; writing – review and editing. **Leonardo Tomei:** Visualization; validation; writing – review and editing; investigation. **Francesca Mori:** Visualization; supervision; writing – original draft; writing – review and editing; validation.

## CONFLICT OF INTEREST STATEMENT

M.G. reports personal fees from Sanofi, Thermo Fisher Scientific. S.B. reports personal fees from Nutricia, Sanofi, and Firma. All other authors declare that they have no conflict of interests to disclose in relation to this paper.

## Supporting information


**Table S1.** Summary of key characteristics of existing published studies on hazelnut oral immunotherapy. IgE, immunoglobulin E; IQR, interquartile range; ITT, intention to treat; mg, milligram; PP, per protocol; SPT, skin prick test; yrs., years.
