# Supplementary material for: Hazelnut oral immunotherapy in children: An Italian single‐center retrospective cohort study
Source: Pediatr Allergy Immunol. 2026 Feb 23;37(2):e70287. doi: 10.1111/pai.70287 (PMC12927672; doi:10.1111/pai.70287)
Supplement: Supplementary file 1 — Table S1. Summary of key characteristics of existing published studies on hazelnut oral immunotherapy. IgE, immunoglobulin E; IQR, interquartile range; ITT, intention to treat; mg, milligram; PP, per protocol; SPT, skin prick test; yrs., years. [file PAI-37-e70287-s001.docx]

|  | Moraly et al. (2020) | Sabouraud et al. (2022)​ | Casanovas et al. (2024)​ | Elizur et al. (2025) |
| --- | --- | --- | --- | --- |
| Study Type | Retrospective, single-center | Retrospective, single-center | Retrospective, single-center | Prospective observational |
| Location | France | Lyon, France | Toulouse, France | Israel |
| Sample Size  median age, (IQR) | 100 patients,  5 yrs (3-9 yrs) | 70 patients,  10 yrs (6.0–13.8) | 88 patients,  8 yrs (5.6–10.8) | 30 patients  ≥4 yrs |
| Desensitization Rate | 34% at 6 months | 51% at 12 months | 52.2% (ITT),  76.7% (PP) at 12 months | 97% |
| Target (desensitized patient)  Maintenance dose | 1635 mg hazelnut protein (8 hazelnuts)    416 mg hazelnut protein (2 hazelnuts) | Individualized dosing | 1490 mg hazelnut protein | 4000 mg hazelnut protein |
| Adverse Reactions | 70% had no side effects 30% had at least one non-severe side effect | 57.1% had mild reactions (2.9% experienced severe systemic allergic reaction, and 1 of them used an epinephrine autoinjector) | 34.2% had mild reactions, 7.4% had anaphylaxis (2.6% required epinephrine at home) | 70% had reactions; none required epinephrine |
| Dropout Rate | Not specified | 21.4%  (mainly due to aversion) | 37.5% (19.3% lost to follow-up, 10.2% personal reasons) | Not reported |
| Predictors of Success | Smaller SPT wheal, lower hazelnut-specific IgE, older age, no cashew allergy | Not specified | Lower baseline IgE (hazelnut, Cor a 9, Cor a 14) predicted success | Not specified |

Table 1 S. Summary of key characteristics of existing published studies on hazelnut oral immunotherapy. IgE: immunoglobulin E; IQR: interquartile range; ITT: intention to treat; mg: milligram; PP: per protocol; SPT: skin prick test; yrs: years.
